# Comparison of the Metabolic Syndrome Risk in Valproate-Treated Patients with Epilepsy and the General Population in Estonia

**DOI:** 10.1371/journal.pone.0103856

**Published:** 2014-07-31

**Authors:** Aleksei Rakitin, Triin Eglit, Sulev Kõks, Margus Lember, Sulev Haldre

**Affiliations:** 1 Department of Neurology and Neurosurgery, University of Tartu, Tartu, Estonia; 2 Department of Internal Medicine, University of Tartu, Tartu, Estonia; 3 Department of Pathophysiology, University of Tartu, Tartu, Estonia; 4 Centre of Translational Medicine, University of Tartu, Tartu, Estonia; 5 Neurology Clinic, Tartu University Hospital, Tartu, Estonia; 6 Internal Medicine Clinic, Tartu University Hospital, Tartu, Estonia; CAEBi, Spain

## Abstract

**Background:**

No study has explored the risk of metabolic syndrome (MS) in patients with epilepsy treated with valproate (VPA) at the population level. The aim of this study was to compare the risk of MS in VPA-treated patients in Estonia to the risk in the general population.

**Methods:**

This study involved 118 patients with epilepsy (63 men, 55 women) who received VPA monotherapy. MS was diagnosed according to the National Cholesterol Education Program Adult Treatment Panel III criteria. Data were compared with the results of a population-based study of the prevalence of MS in the same geographic region (N = 493; 213 men, 280 women).

**Results:**

In the multiple logistic regression analysis, after adjustment for age and sex, the risk of MS in VPA-treated patients was not increased compared to the control subjects (odds ratio [OR] = 1.00; 95% confidence interval [CI], 0.59–1.68). VPA-treated patients had higher serum insulin concentrations than control subjects, independent of body mass index (BMI). A positive association was found between MS development and BMI (OR = 1.47; 95% CI, 1.25–1.73) in VPA-treated patients, but there were no associations with the VPA dosage or the homeostasis model assessment-estimated insulin resistance (HOMA-IR) index. In control subjects, BMI and HOMA-IR had similar predictive abilities for MS occurrence. In VPA-treated patients, the predictive ability of the HOMA-IR index was significantly lower than that of BMI, with areas under the receiver operating characteristic curves of 0.808 and 0.897 (P = 0.05), respectively.

**Conclusions:**

The risk of MS is not increased among VPA-treated patients with epilepsy in Estonia compared to the general population. The HOMA-IR index likely has a lower predictive ability for MS in VPA-treated patients compared to its predictive ability in the general population.

## Introduction

Valproate (VPA) is one of the most frequently used antiepileptic drugs worldwide [1]. It is currently widely applied for other indications as well, such as bipolar disorder and migraine prophylaxis [2]. In many cases, the duration of treatment may be long, which emphasizes the importance of the long-term safety of the drug. One of the most common side effects of VPA treatment is weight gain, which occurs in about half of patients and is associated with important metabolic and endocrine abnormalities [3]. VPA treatment is also associated with elevated serum insulin levels and changes in the levels of triglycerides (TG) and high-density lipoprotein cholesterol (HDL-C) [4].

In 1988, Reaven described metabolic syndrome (MS) as a cluster of metabolic conditions, including glucose intolerance, dyslipidemia, central obesity, and hypertension, which are major risk factors for cardiovascular and cerebrovascular diseases [5]. The worldwide prevalence of MS in the adult population has been reported to be about 24.7–28.8% [6]–[10]. In a recent population-based study, the estimated prevalence of MS in Estonia was 27.9% [11]. Although many studies have explored the problem of obesity in VPA-treated patients, the presence of MS among them has received little attention. Verrotti at al. [12] found that the prevalence of MS in children and adolescents treated with VPA who became obese was similar to that observed in otherwise healthy overweight subjects. However, another study found that obese patients with epilepsy who were treated with VPA were at higher risk of developing MS than obese control subjects [13].

No study to date has explored the risk of MS in VPA-treated patients at the population level. Thus, the aim of our study was to evaluate the prevalence of MS and its components in VPA-treated patients with epilepsy in Estonia, compared to the general population.

## Methods

### Subjects

The study was carried out in the Departments of Neurology and Internal Medicine, Tartu University Hospital, Estonia, between 1 January and 31 December 2012. This hospital is a tertiary referral center for southern Estonia. Distributions of the ethnic groups and the socioeconomic status of inhabitants are similar among the various counties in southern Estonia.

Using the prescription database of the Estonian Health Insurance Fund, patients with epilepsy from six southern Estonian counties who were prescribed VPA in 2011 were identified. Exclusion criteria for patients with epilepsy were as follows: age ≥18 years; VPA monotherapy for >3 months; polytherapy with other antiepileptic drugs; pregnancy; and severe physical or mental disability. In particular, patients who lived at nursing homes or who were dependent at home or outside the home were excluded. Only patients who were able to engage in normal occupational and social activities, despite minor physical or mental deficits, were included in the study. Patients with endocrine disturbances, such as diabetes or thyroid dysfunction, were not excluded.

The control group comprised subjects who participated in a population-based cross-sectional multicenter study of MS prevalence conducted in southern Estonia between November 2008 and May 2009. Control subjects were adults who were randomly selected from four general practices. An invitation letter about the study was sent to each participant. The total response rate was 53.2% (493 control subjects). The control subjects were representative of the general Estonian population in terms of age and gender.

### Collection of anthropometric and laboratory data

The first and second author, respectively, interviewed and clinically examined all patients and control subjects. The medical histories of participants were documented during the evaluation meeting. Concomitant diseases of relevance to the study, including known endocrinopathies, lipid metabolism disorders, and vascular diseases, were noted. Blood pressure (BP), waist circumference, weight, and height were measured, and body mass index (BMI) was calculated as weight (in kg) divided by height squared (in m^2^). Blood samples were obtained in the morning (between 08:00 and 11:00) after an overnight fast (≥10 h) for the analysis of serum insulin, HDL-C, TG, and plasma glucose concentrations. Patients' serum VPA concentrations were also measured.

During face-to-face interviews, patients' epilepsy diagnoses were re-evaluated, and the date of the last seizure was recorded. Seizures and epileptic syndromes were classified based on the recently proposed International League against Epilepsy classification [14]. In patients with classical idiopathic generalized epilepsy syndromes resulting directly from a known or presumed genetic defect(s), the etiology of epilepsy was classified as “genetic”. The date of initiation, duration, and current dosage of VPA treatment were recorded. Patients were asked whether they had noticed any change in body weight after initiation of VPA treatment. The Ethics Review Committee on Human Research of Tartu University approved the study. All participants provided written informed consent.

### Assays

Plasma glucose levels were measured by the hexokinase method. HDL-C and TG concentrations were measured by an enzymatic colorimetric assay. VPA concentrations were measured by fluorescence polarization assay (COBAS INTEGRA 800 Plus Analyzer; Roche, Basel, Switzerland). In control subjects, plasma insulin concentrations were measured by a chemiluminescent assay (Immulite 2000 Analyzer; Siemens Healthcare Diagnostics, Deerfield, IL, USA), whereas in patients, they were measured by an electrochemiluminescent assay (COBAS 6000 Analyzer; Roche). To compare plasma insulin concentrations between cohorts, the bias between the two measurement assays was calculated according to the Clinical and Laboratory Standards Institute's guidelines [15].

### Definition of metabolic syndrome

MS was diagnosed based on the presence of at least three of the following National Cholesterol Education Program Adult Treatment Panel III criteria [16]: waist circumference ≥102 cm in men and ≥88 cm in women, BP≥130/85 mmHg or antihypertensive medication use, fasting glucose concentration ≥5.6 mmol/L or previously diagnosed diabetes, TG concentration ≥1.7 mmol/L or lipid-regulating medication use, and HDL-C concentration <1.03 mmol/L in men and <1.3 mmol/L in women or drug treatment for reduced HDL-C. Insulin resistance (IR) was estimated by the homeostasis model assessment-estimated insulin resistance (HOMA-IR) index, calculated with the following equation: fasting glucose (mmol/L)×fasting insulin (mU/L)/22.5. Patients with BMIs≥25 kg/m^2^ were categorized as overweight.

### Statistical analysis

Descriptive analytical methods, such as the calculation of means, standard deviations (SDs), medians, and interquartile ranges (IQRs), were used for continuous variables, depending on the distribution. Age- and sex-adjusted linear regression models were used to compare anthropometric and laboratory data between VPA-treated patients and control subjects. Multiple logistic regression analysis was performed to identify factors associated with MS development in VPA-treated patients and control subjects. The final model included only significant factors. A receiver operating characteristic (ROC) analysis was conducted to evaluate whether BMI and HOMA-IR had similar predictive abilities for MS in both cohorts. The prevalence of MS in VPA-treated patients was calculated by the indirect method of standardization, considering the age and sex distribution of the control group as the standard. Odds ratios (ORs) and 95% confidence intervals (95% CIs) are reported. The R software package (The R Foundation for Statistical Computing; version 2.15.1) was used for statistical analyses. Differences with P≤0.05 were considered statistically significant.

## Results

**Figure 1:**
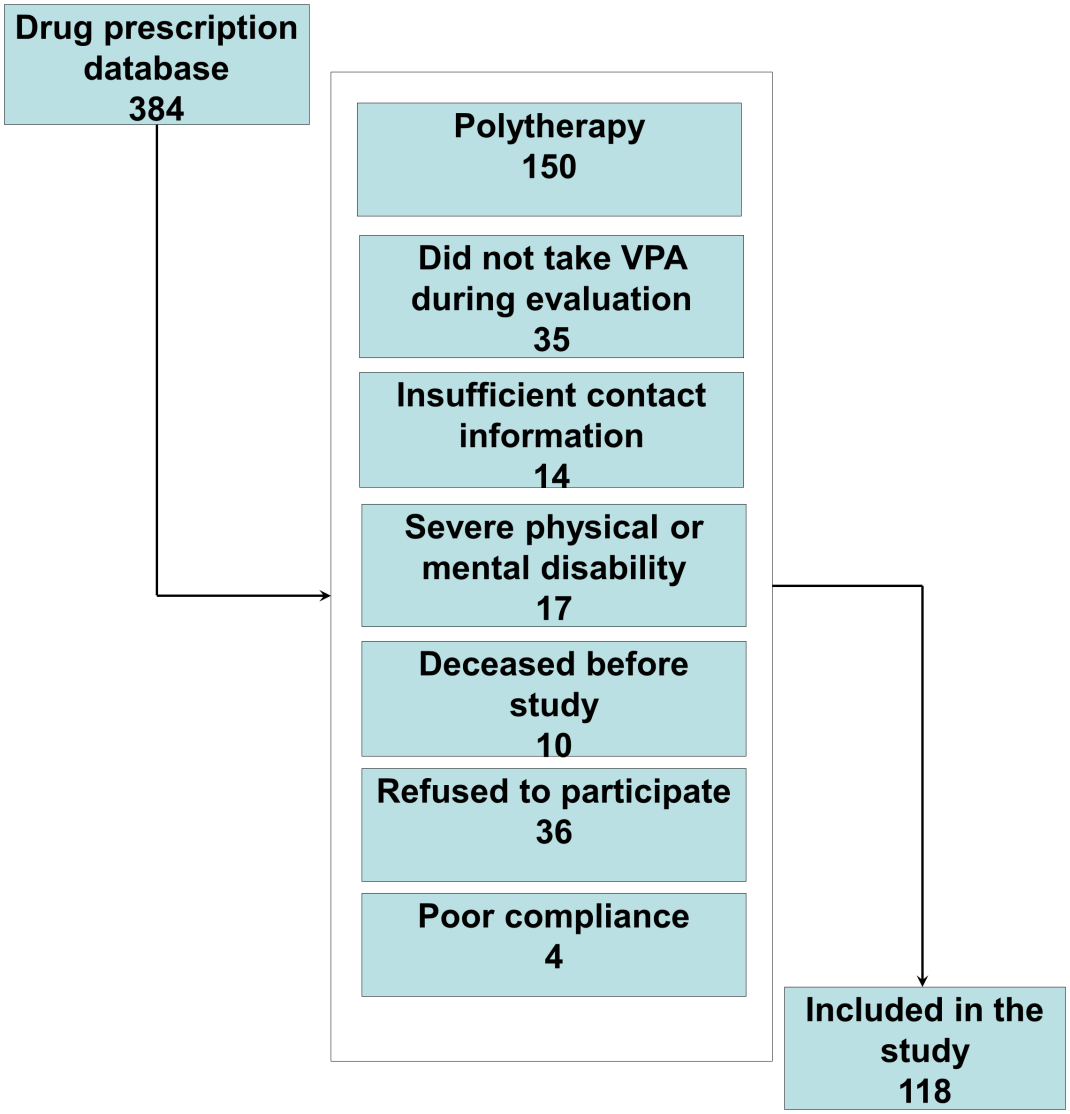
Flowchart for the inclusion of valproate-treated patients with epilepsy.

A total of 384 patients (206 men, 178 women) with epilepsy diagnoses who had received VPA treatment were identified. Employing the data collection methods described above, 122 patients (64 men, 58 women) who met the inclusion criteria and agreed to participate were included in the study. Subsequently, four patients were excluded because of low serum VPA levels measured during the evaluation visit (Figure 1). The final study sample comprised 118 adult patients with epilepsy (63 men, 55 women) and 493 control subjects (213 men, 280 women). Median ages of the patients and control subjects were 32 years (IQR, 24–45 years) and 47 years (IQR, 35–59 years), respectively. In recently published studies, the mean reported increase of BMI after initiation of VPA treatment in adults was around 2.5 kg/m^2^ [17]–[22]. The power of the present study for detecting a BMI difference of 2.5 kg/m^2^ with a SD of 5 is >90%. The clinical characteristics of the patients are given in Table 1.

**Table 1:** Clinical characteristics of patients with epilepsy who received valproate treatment.

	No. of patients	Age (years)	Patients with seizures during last year	Seizure type			Epilepsy etiology			Daily VPA dose (mg/d)	Serum VPA concentration (µg/ml)
				G	L	U	Gen	Str/met	U		
Male	63(53%)	36.1±15.3	24(20%)	33(28%)	22(19%)	8(7%)	33(28%)	15(13%)	16(14%)	1027±487	58.6±29.2
Female	55(47%)	37.1±16.0	30(25%)	38(32%)	14(12%)	3(3%)	36(31%)	5(4%)	13(11%)	894±386	63.7±32.4
All	118(100%)	35.6±16.3	54(46%)	71(60%)	36(31%)	11(9%)	69(58%)	20(17%)	29(25%)	964±446	61.0±30.7

Values are expressed as means (%) ± standard deviations, except for the number of patients, seizure type, and epilepsy etiology.

No, number; G, primarily generalized seizure type; L, localization-related seizure type; U, unknown; Gen, genetic; Str/met, structural/metabolic; VPA, valproate.

Seventy-one (60.2%) patients had generalized and 36 (30.5%) had focal seizures; seizure type was unclear in 11 (9.3%) patients. Sixty-four (54.3%) patients had been seizure-free for at least 1 year. The median duration of VPA treatment was 6 years (IQR, 2.5–10.0 years).

The crude prevalence of MS in adult patients with epilepsy who received VPA monotherapy was 20.3% (95% CI, 13.7–28.9%); this prevalence was 22.2% (95% CI, 13.1–34.8%) in men and 18.2% (95% CI, 9.5–31.4%) in women. The prevalence of MS in VPA-treated patients, weighted by the age and sex distribution of the control cohort, was 25.8% (95% CI, 18.4–34.8%). The prevalence of MS in the control group was 27.9% (95% CI, 24.0–32.1%). In the final multiple logistic regression, after adjustment for age and sex, the risk of MS in VPA-treated patients was not increased (OR = 1.00; 95% CI, 0.59–1.68; P = 0.998). However, a larger proportion of VPA-treated patients had abnormal diastolic BP (≥85 mmHg) compared with control subjects (OR = 1.86; CI 1.15–3.03; P = 0.011; Table 2).

**Table 2:** Logistic regression model for metabolic syndrome and its components in patients treated with valproate compared to control subjects.

Component	OR	95% CI	P
Waist circumference	0.75	0.47–1.22	0.248
men, >102 cm	0.89	0.45–1.75	0.741
women, >88 cm	0.64	0.32–1.26	0.190
HDL-C	0.95	0.57–1.59	0.851
men, <1.03 mmol/L	1.31	0.63–2.69	0.467
women, <1.30 mmol/L	0.76	0.36–1.63	0.490
Triglycerides ≥1.7 mmol/L	1.34	0.80–2.24	0.267
Fasting glucose ≥5.6 mmol/L	0.78	0.46–1.31	0.352
Systolic blood pressure ≥130 mmHg	1.54	0.91–2.60	0.112
Diastolic blood pressure ≥85 mmHg	1.86	1.15–3.03	0.011
Metabolic syndrome	1.00	0.59–1.68	0.998

Odds ratios for metabolic syndrome and its components in valproate-treated patient cohort compared controls adjusted for age and sex.

The clinical and biochemical characteristics of patients with and without MS are shown in Table 3. Most (58.3%) patients with epilepsy and MS had three components of the syndrome, 29.2% had four components, and 12.5% had all five components. In control subjects with MS, these percentages were 56.4%, 31.5%, and 12.1%, respectively. Arterial hypertension (83.3%), abdominal obesity (83.3%), and increased TG level (75.0%) were the most common abnormalities in patients with epilepsy. In the control subjects, the most common MS components were arterial hypertension (93.6%), abdominal obesity (91.4%), and impaired glucose metabolism (71.4%).

**Table 3:** Characteristics of subjects receiving valproate monotherapy with and without metabolic syndrome.

Characteristic	Metabolic syndrome n = 24	No metabolic syndrome n = 94	P
Sex (male/female)	14/10	49/45	<0.0001
Age (years)	44.6±13.6	34.4±15.4	<0.001
Waist circumference (cm)	106.2±10.4	82.3±13.5	<0.0001
Men	104.0±10.1	88.7±12.3	<0.0001
Women	109.6±10.5	75.6±11.3	<0.0001
Body mass index (kg/m^2^)	32.5±5.2	24.7±4.0	<0.0001
Men	30.5±4.3	25.7±3.7	<0.0001
Women	35.5±5.3	23.7±4.1	<0.0001
HOMA-IR	3.90±2.3	1.85±1.3	<0.0001
Men	3.8±2.4	2.1±1.4	<0.01
Women	4.0±2.1	1.6±1.1	<0.001
Systolic blood pressure (mmHg)	144.2±23.8	120.5±14.8	<0.0001
Diastolic blood pressure (mmHg)	91.3±11.6	78.5±11.5	<0.0001
Triglycerides (mmol/L)	2.4±1.3	1.1±0.5	<0.0001
HDL-C - men (mmol/L)	1.1±0.3	1.4±0.4	<0.0001
HDL-C - women (mmol/L)	1.2±0.2	1.8±0.5	<0.0001
Fasting glucose (mmol/L)	5.7±1.2	5.0±0.5	<0.001
Fasting insulin (mU/L)	16.0±10.1	8.2±5.5	<0.0001
Daily VPA dose (mg)	1060±406	939±454	>0.05
VPA treatment duration (months)	128±85	70±58	<0.01

Data presented as means ± standard deviations.

VPA, valproate; HOMA-IR, homeostasis model assessment of insulin resistance.

BMI did not differ between VPA-treated patients and control subjects (P = 0.295); however, VPA-treated patients had higher fasting serum insulin levels compared to control subjects (P<0.0001). The HOMA-IR index was higher in patients than in control subjects (P = 0.0001). These differences persisted when only normal-weight subjects from both cohorts were compared (P = 0.010 for insulin and P = 0.031 for HOMA-IR). Fasting plasma glucose concentrations tended to be lower in patients than in control subjects, although this difference was not significant (P = 0.169). The HDL-C and TG levels were similar in patients and control subjects (Table 4).

**Table 4:** Anthropometric and laboratory data in the patients and control subjects.

	Patients	Control subjects	P
BMI (kg/m^2^)	26.38±5.34	28.18±6.16	0.295
Fasting glucose (mmol/L)	5.18±0.78	5.43±0.82	0.169
Fasting insulin (mU/L)	9.10±8.51	6.35±5.52	<0.0001
HOMA-IR	2.21±2.01	1.60±1.4	<0.001
HDL-C (mmol/L)	1.51±0.49	1.53±0.45	0.581
Triglycerides (mmol/L)	1.34±0.91	1.31±0.72	0.375

Data presented as means ± standard deviations.

BMI, body mass index; HOMA-IR, homeostasis model assessment of insulin resistance.

P values were calculated after comparison of parameters between cohorts using multiple linear regression model adjusted for age and sex.

**Figure 2:**
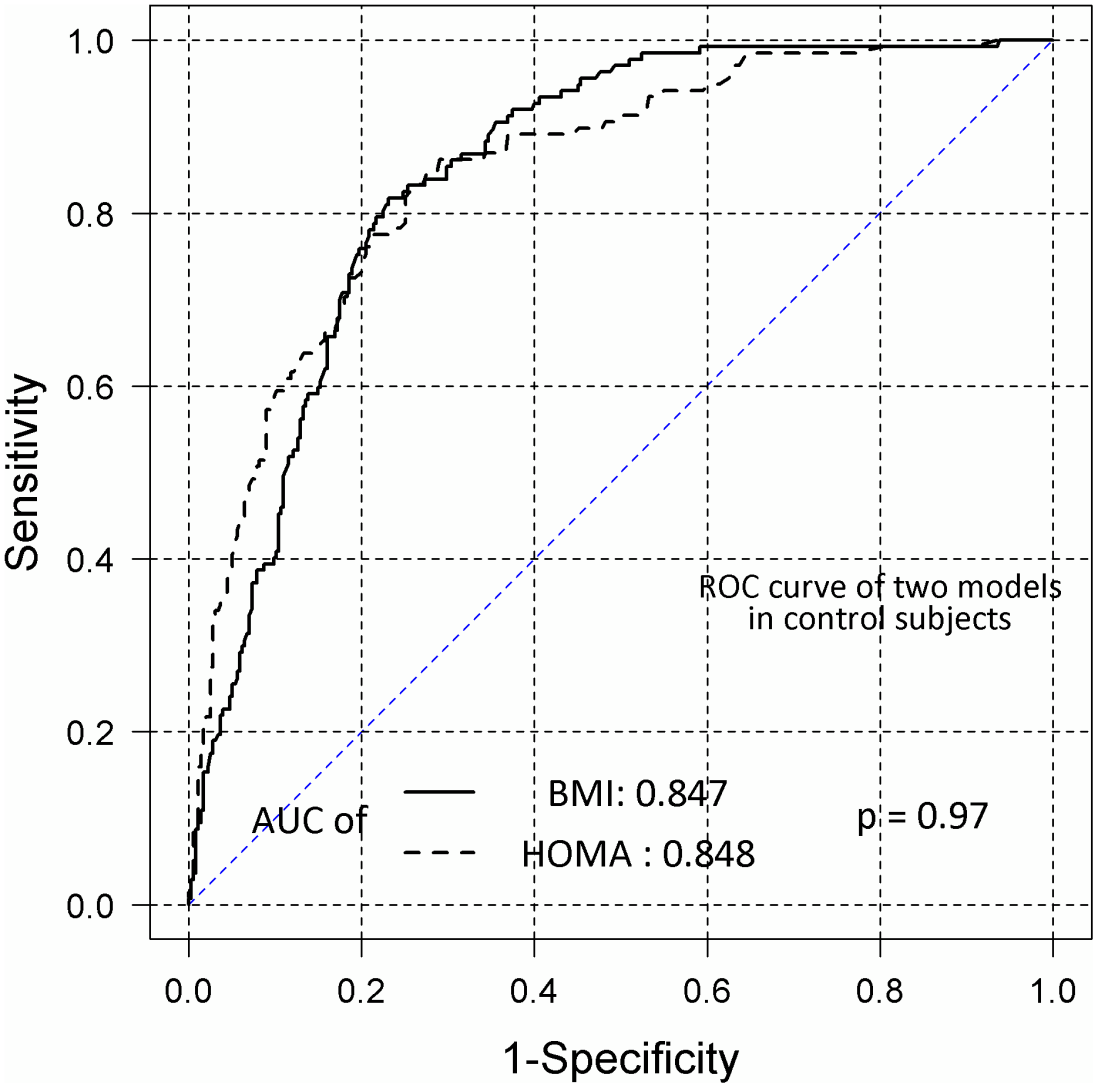
Receiver operating characteristic (ROC) curves of the homeostasis model assessment-estimated insulin resistance (HOMA-IR) and body mass index (BMI) values in control subjects. BMI and HOMA-IR curves were obtained by logistic regression to determine the probability of metabolic syndrome (MS) in control subjects. AUC, area under the curve.

**Figure 3:**
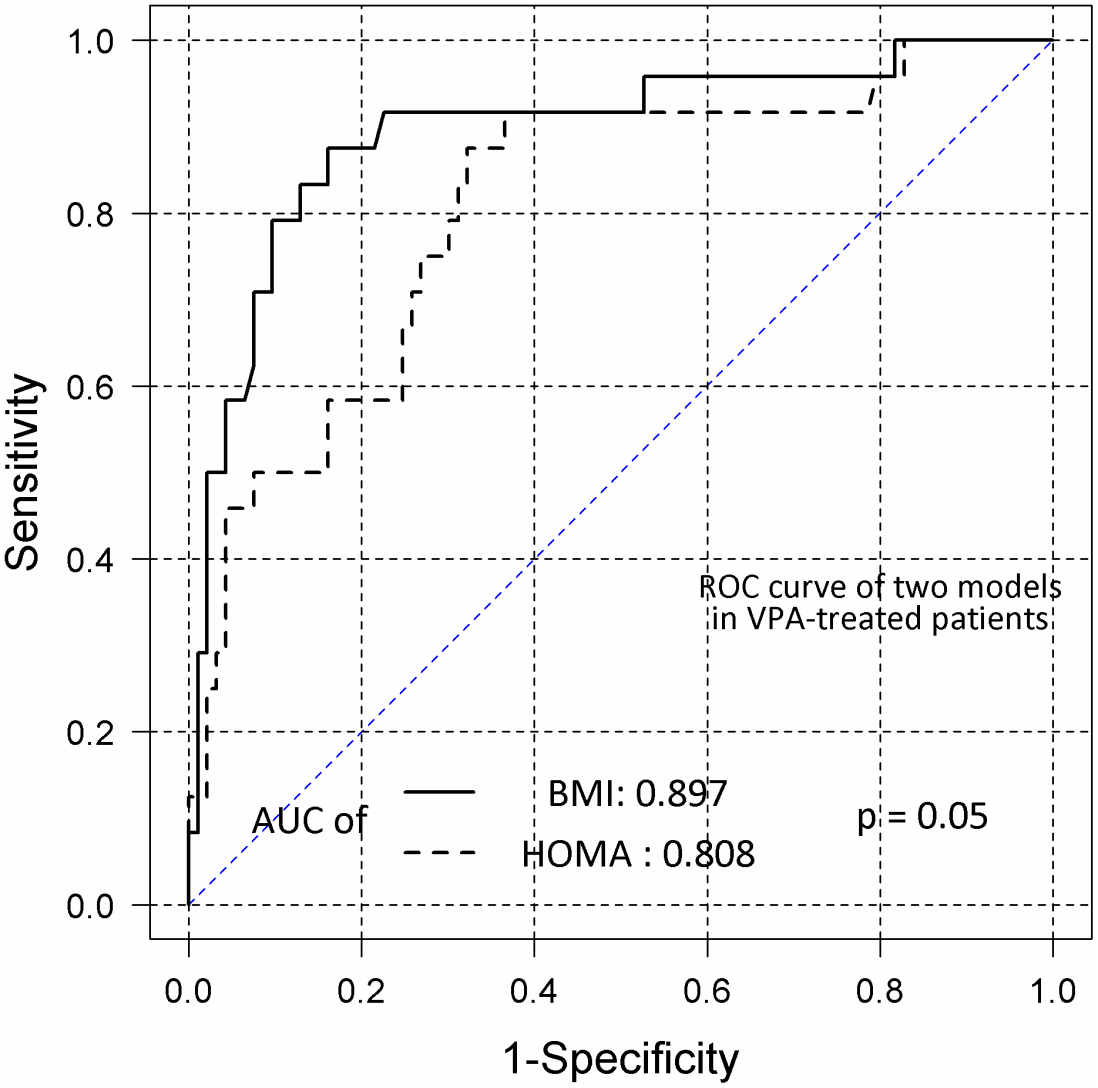
Receiver operating characteristic (ROC) curves of the homeostasis model assessment-estimated insulin resistance (HOMA-IR) and body mass index (BMI) values in valproate (VPA)-treated patients. BMI and HOMA-IR curves were obtained by logistic regression to determine the probability of metabolic syndrome (MS) in patients treated with VPA. AUC, area under the curve.

Logistic regression analysis showed no significant correlation between MS development in VPA-treated patients and any clinical characteristic, including age, sex, VPA dosage, HOMA-IR, epilepsy etiology, seizure occurrence in the last year, and subjectively reported weight gain during VPA treatment. Positive correlations were found between MS development and BMI (OR = 1.47; 95% CI, 1.25–1.73). Longer durations of VPA treatment tended to increase the risk of MS, with borderline statistical significance (OR = 1.01; 95% CI, 1.0–1.02). In overweight VPA-treated patients, the predictors of MS were HOMA-IR (OR = 2.56; 95% CI, 1.40–4.70) and age (OR = 1.07; 95% CI, 1.01–1.13). In control subjects, MS development was correlated positively with HOMA-IR (OR = 2.00; 95% CI, 1.61–2.49), BMI (OR = 1.18; 95% CI, 1.12–1.24), and age (OR = 1.04; 95% CI, 1.02–1.06). ROC analysis showed that BMI and HOMA-IR similarly predicted MS occurrence in control subjects, with areas under the ROC curve (AUCs) of 0.847 and 0.848 (P = 0.97), respectively (Figure 2). The predictive ability of HOMA-IR was lower than that of BMI in VPA-treated patients, with AUCs of 0.808 and 0.897 (P = 0.050), respectively (Figure 3).

## Discussion

In the past decade, increasing numbers of studies have examined the adverse effects of VPA (e.g., weight gain, hyperinsulinemia, and endocrine abnormalities), reflecting growing concern among physicians and patients who use this drug [23]. However, few studies have explored the potential relationship between prolonged VPA treatment and MS in persons with epilepsy [12], [13]. For the first time, we demonstrated that the prevalence of MS in adult patients with epilepsy who received VPA treatment was 25.8%, which did not differ from the results of a population-based study [11]. Because subjects in both cohorts were drawn from the same population over the same extended time period, differences in genetic background and nutritional habits were probably minimal, which makes the results even more reliable.

The tendency of patients with epilepsy on long-term VPA treatment to be overweight is generally recognized in the medical community [19]. However, most studies examining weight gain and abdominal obesity during VPA treatment have been incident studies [17], [20], [21], [24] or have used selected control subjects for comparison [22]. Our results show that BMI is not higher in these patients than in the general population. Our study design is the main reason for these results, as patients whose VPA treatment was discontinued due to side effects were not included in our cohort. This design likely resulted in negative selection bias against VPA-related side effects, such as weight gain. Nevertheless, the study reflects the true clinical practice of medical VPA usage and the management of VPA-related side effects in patients with epilepsy.

A long duration of VPA therapy tended to increase the risk of MS, but we found no correlation between daily VPA dosage and MS development. These results are consistent with previous reports showing a positive correlation between VPA treatment duration and significant weight gain, and no influence of VPA dosage [23]. Several studies reported that VPA-treated patients exhibited hyperinsulinemia and a tendency toward lower plasma glucose levels (reviewed by [3]), findings that were confirmed by our results. The observation of these changes in both obese and normal-weight patients suggests that hyperinsulinemia is the leading factor for weight gain and MS development during VPA treatment [25].

Lipid metabolism findings in VPA-treated patients have been inconsistent. Some studies have associated VPA treatment with decreased HDL-C [4] and increased TG [4], [19],[22] levels, whereas others have found no effect of VPA treatment on lipid metabolism [13], [26]. One possible explanation is that VPA has no direct influence on TG or HDL-C metabolism, in contrast to its effect on insulin. Thus, the unfavorable changes in lipid levels probably occur during the development of MS, the prevalence of which was not increased among VPA-treated patients in our study. The diversity of previously reported data on changes in lipid levels during VPA treatment is probably related to differences in the subject selection methods. Specifically, the lipid profiles are probably more unfavorable in cohorts with higher proportions of VPA-treated patients with MS.

Previous studies have paid little attention to the important side effects of prolonged VPA treatment, such as a tendency towards elevated BP [4], [13]. This characteristic can be explained by the increased serum insulin levels in VPA-treated patients, which can lead to elevated sympathetic activity [27]. The increased proportion of VPA-treated patients with high BP, in the absence of differences in the prevalence of other MS components, suggests that the tendency towards hypertension is a direct effect of VPA treatment, probably due to hyperinsulinemia. Although MS did not occur more frequently in our VPA-treated patients, the risks of cardiovascular and cerebrovascular diseases may be increased, given than hypertension is the leading cause of these conditions.

The pathophysiology of MS remains unclear, but many of its features are associated with IR, which is typically defined as decreased sensitivity or responsiveness to the metabolic action of insulin [28]. The gold standard method for assessment of IR is the hyperinsulinemic-euglycemic clamp technique originally developed by DeFronzo et al. [29]. As this method is complex and unsuitable for epidemiological investigations, several surrogate indices, such as the HOMA-IR index, have been developed.

Previous studies using the HOMA-IR index have reported the presence of IR in patients receiving VPA treatment [3], [13], [22], [23], [26]. However, an increased HOMA-IR index in these patients most likely reflects hyperinsulinemia, which can occur in obese and normal-weight patients. Pylvänen et al. [25] first raised this issue and suggested that VPA interferes with insulin degradation in the liver, resulting in higher insulin concentrations in the peripheral circulation. In this case, an increased HOMA value probably does not reflect reduced sensitivity to insulin. Indeed, we found significantly higher HOMA indices in VPA-treated patients than in control subjects, although the prevalence of MS was similar in both cohorts. ROC analyses of the predictive abilities of BMI and HOMA-IR for MS suggested that HOMA-IR was an inferior predictor of MS in VPA-treated patients compared to the general population. These results allow us to speculate that previous studies applying the HOMA-IR index in VPA-treated patients have probably overestimated the occurrence of IR.

The primary limitation of our study was the difference in age distribution between cohorts. Age is an important determinant of MS [30]. Patients with epilepsy were younger than control subjects, as VPA is used mainly for the treatment of generalized epilepsy, which usually manifests in children and young adults. Because they were generally older, the subjects in the control group might have been more likely to develop MS, causing a negative bias against MS in the patient cohort. To overcome this limitation, we used age- and sex-adjusted logistic regression analysis, as well as indirect standardization.

Physical inactivity is another important risk factor for MS [30]. People with epilepsy tend to participate less often in physical activities, as well as to have lower levels of endurance and flexibility, compared to subjects without epilepsy [31]. However, the physical activity levels of the patients and control subjects were not assessed in the current study, which constitutes another important limitation. Further studies should also compare the risk of MS in people with epilepsy who take different antiepileptic drugs as monotherapy. Considering these limitations, the results of this study should be interpreted carefully. Given that the study design focused on prevalence, the findings cannot be used to indicate whether VPA causes weight gain. Nevertheless, we observed that patients with epilepsy who received VPA treatment were not more overweight than other people in our population.

In conclusion, we found that the risk of MS and increased body weight was not greater in patients with epilepsy who had received VPA treatment than in the general population. However, VPA-treated patients had higher serum insulin concentrations, independent of BMI, suggesting that hyperinsulinemia is not a consequence of obesity. HOMA-IR had a lower predictive ability for MS in patients who received VPA treatment compared to the general population.
